# Knowledge, Attitude, and Practice of Physicians Regarding Vaccinations in Yerevan, Armenia: A Case Study of HPV

**DOI:** 10.3390/vaccines9101188

**Published:** 2021-10-15

**Authors:** Arman R. Badalyan, Marine Hovhannisyan, Gayane Ghavalyan, Mary M. Ter-Stepanyan, Rory Cave, Jennifer Cole, Andrew W. K. Farlow, Hermine V. Mkrtchyan

**Affiliations:** 1 Department of Epidemiology, Faculty of Public Health, Yerevan State Medical University after M. Heratsi, Yerevan 0025, Armenia; armanfcj@yahoo.com (A.R.B.); mhovannis@yahoo.com (M.H.); gayane_ghavalyan@yahoo.com (G.G.); tsmary@mail.ru (M.M.T.-S.); 2 School of Biomedical Sciences, University of West London, London W5 5RF, UK; rory.cave@uwl.ac.uk; 3 Department of Health Studies, Royal Holloway University of London, Egham Hill, Egham TW20 0EX, UK; Jennifer.cole@rhul.ac.uk; 4 Oxford Martin School, University of Oxford, Broad St, Oxford OX1 3BD, UK; andrew.farlow@ndm.ox.ac.uk; 5 Oxford in Berlin, Museum für Naturkunde, 10115 Berlin, Germany

**Keywords:** immunization, vaccine confidence, vaccine hesitancy, HPV, cervical cancer, Gardasil

## Abstract

This paper highlights the low levels of vaccine coverage and high levels of reported vaccination hesitancy in Yerevan, Armenia, that present profound challenges to the control of disease through routine vaccination programmes. We draw on investigations of hesitancy towards the introduction of new vaccines, using the Human Papillomavirus (HPV) vaccine Gardasil as a case study, to interrogate underlying challenges to vaccine acceptance. We analyse primary data from the introduction of Gardasil, first used in Armenia in 2017, to investigate how levels of medical knowledge amongst physicians in 20 health facilities in Yerevan, Armenia, regarding vaccine science influence attitudes towards the introduction of a newly developed vaccine. A questionnaire-based cross-sectional study was completed by 348 physicians between December 2017 and September 2018. The responding physicians displayed a respectable level of knowledge and awareness regarding vaccination with respect to some characteristics (e.g., more than 81% knew that HPV infection was commonly asymptomatic, 73% knew that HPV infection was implicated in most cervical cancers, and 87% knew that cervical cancer is the most prevalent cancer amongst women) but low knowledge and poor understanding of other key issues such as the age at which women were most likely to develop cervical cancer (only 15% answered correctly), whether or not the vaccine should be administered to people who had already been infected (27% answered correctly) and whether sexually active young people should be treated for infection before vaccination (26% answered correctly). The study suggests that the drivers of vaccine hesitancy are complex and may not be consistent from vaccine to vaccine. The Armenian healthcare sector may need to provide additional training, awareness-raising and educational activities alongside the introduction of new vaccines to improve understanding of and trust in vaccination programmes.

## 1. Introduction

Armenia has consistently displayed lower levels of vaccine confidence than surrounding countries and Europe as a whole [1,2,3], with Armenians showing low levels of trust in vaccines being safe, effective and/or important [1]. This has significant implications for control and prevention of vaccine-controllable diseases and presents a challenge in that the frequency of novel diseases, for which new vaccines need to be developed, is predicted to increase during the 21st century [4], risking further pandemics such as that caused by COVID-19 [5]. In this paper, we use the introduction of the HPV vaccine Gardasil in Yerevan, Armenia as a case study for understanding vaccine hesitancy in Armenia more broadly, particularly with regard to the introduction of new vaccines, and for shedding light on some key underlying reasons for hesitancy. A new national vaccination programme for the human papillomavirus (HPV) has been available since 2017 but coverage in Armenia is low (10% in the target population in 2020, compared with 84% in the UK, for example [6]). We use this to identify potential inventions that may improve vaccine acceptance and confidence, thus enhancing the uptake of vaccines for HPV, and which may be generalizable to other diseases, using the HPV vaccine to conceptualise vaccine hesitancy in Armenia. Thus, HPV offers a case study that may inform the roll-out of other national vaccination programmes. The primary data collection has been reviewed with reference to two recent market research polls that have interrogated vaccine hesitancy in Armenia [7,8].

Human papillomaviruses (HPVs) are a group of viruses belonging to the family of *Papillomaviridae* that affect epithelial tissue, the layer or layers of cells that form the covering of most internal and external surfaces of the body and its organs. More than 150 types of HPV have been identified [9]. In women, HPV infection may cause cervical, vaginal, vulvar, anal, and oropharyngeal cancers; in males, it may cause anal, penile, and oropharyngeal cancers [10,11,12].

Invasive cervical cancer (ICC) is one of the leading causes of cancer in women and, according to 2018 global estimates, results in approximately 570,000 new cases and 311,000 deaths annually [13]. In the United States, around 4000 women die from cervical cancer each year, with African Americans and women from poorer backgrounds having a much higher rate of mortality [14]. Globally, cervical cancer is the fourth most common cancer among women. The burden faced by low- and middle-income countries is significantly higher than in high-income regions of the world [15]. The estimated age-standardised incidence of cervical cancer in 2018 averaged 13.1 per 100,000 women globally but varied from less than 2 up to 75 per 100,000 women [13], depending on the country.

In Armenia, the cumulative incidence rate of ICC has increased from 9.5 (1985) to 16.6 (2017) per 100,000 women. Annually, about 120–130 women die from cervical cancer in Armenia (7.4/100,000 in 2016). About 50% of women with ICC are not diagnosed until the third and fourth stages of cancer, when the treatment outcome is significantly less certain [16,17].

Currently, there are three HPV vaccines available, bivalent (HPV 16 and 18), quadrivalent (HPV 6, 11, 16, and 18), and 9-valent (6, 11, 16, 18, 31, 33, 45, 52, and 58), which can protect against up to nine of the most prevalent HPV genotypes associated with cancer and genital warts [18]. Of these, the quadrivalent is the most common in Armenia. Globally, HPV vaccines have been extremely successful from both a scientific and a clinical perspective. There is strong evidence of population-level impact on the circulation of HPV infection, incidence of high-grade cervical cancer and cases of genital warts following the introduction of national vaccination programmes [19,20].

Despite recommendation for routine HPV vaccinations from the World Health Organization (WHO), some countries nevertheless remain hesitant about vaccinating their young people against HPV [21]. Armenia is one of these and the vaccine hesitancy shown with regard to HPV vaccination provides warnings of challenges that may also hinder other vaccination programmes and thus the management of other vaccine-controllable diseases [22].

Since the quadrivalent vaccine Gardasil was introduced in Armenia in December 2017 [23], uptake has been slow, with resistance from the medical community as well as from the public. A study assessing the knowledge, attitudes, and practices (KAP) of physicians regarding HPV and vaccination against HPV, particularly with Gardasil, in Yerevan, Armenia, conducted between Dec 2017 and Sep 2018 provides baseline understandings of some causes of vaccine hesitancy and highlights some issues that may present similar barriers to the uptake of vaccines developed to combat COVID-19. The survey results reveal, in addition, that the term “vaccine hesitancy” needs to be unpacked further, as there are degrees or shades of hesitancy that range from near-acceptance to out-right rejection. These are important if we are to fully understand the challenges of introducing a new vaccine to a hesitant population.

## 2. Materials and Methods 

Between December 2017 and September 2018, a questionnaire-based, cross-sectional, quantitative study was conducted in 20 Armenian healthcare facilities to identify respondents’ awareness of and attitudes toward HPV-related cervical cancer and the Gardasil vaccine. The questionnaire did not contain questions specifically related to practice, as this is known to be sub-optimal; the aim of the study was to determine the reasons why. The questionnaire was adapted from a survey previously used to determine the knowledge and attitude of registered nurses towards HPV and HPV vaccines in the USA [24]. This was considered sufficiently generic to not require amendment to the local context. The questionnaire was administered to physicians at 20 out of 36 government-run healthcare facilities, selected at random from those available, in Yerevan; this was the number required to ensure sufficient staff to return a representative sample size, calculated using the Estimate-proportion n = z^2^*pq/d^2^ formula [25].

**Figure 1:**
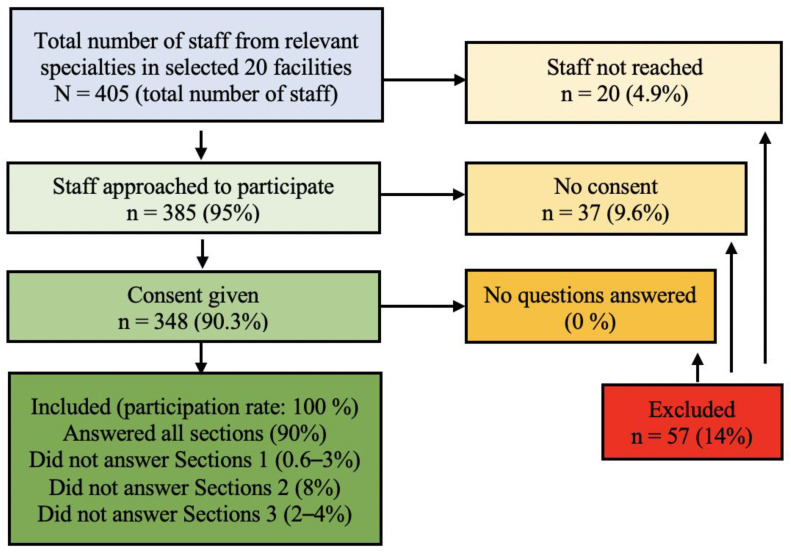
Flow chart of staff recruitment.

The inclusion criteria for the study respondents were fully qualified medical staff from four medical specialities considered most likely to have direct interaction with HPV-infected patients or with patients eligible for HPV vaccination, namely, paediatricians, family-practice doctors, gynaecologists, and oncologists. Exclusion criteria were student doctors, nurses, residents, and doctors from other specialities. This yielded a possible survey size of 405 physicians across the 20 facilities. Trained researchers from Yerevan State University School of Public Health visited each of the healthcare facilities included and personally invited physicians to participate in the study. Of the 405 eligible staff, 385 (95%) were approached in person to participate (the remaining 20 were not available on any of the days the researchers visited) of whom 348 (90.3%) gave consent and completed the survey. The survey was conducted by face-to-face interview in each physician’s usual place of work. This is above the number required to be considered a representative sample (Figure 1).

Data were collected on the socio-demographic characteristics of the respondents (gender, age, and length of service within the healthcare profession). The questionnaire also addressed the respondents’ existing knowledge and attitudes across four areas: (1) knowledge/information about HPV; (2) knowledge/information about the HPV vaccine; (3) attitude towards the HPV vaccine; (4) attitude towards other vaccines. Respondents could answer the survey questions “True”, “False” or “I don’t know”, numerically coded 1, 2, and 88, respectively.

### 2.1. Statistical Analysis

Chi-square analyses were used for the categorical variables which compared the age groups of respondents, their lengths of experience as doctors, and the fields they specialised in. For all the analyses conducted in the study, an association of *p* < 0.05 was considered to be statistically significant. The analyses were performed using the SPSS software (Version 16.0). 

A hierarchy heatmap on the physicians’ knowledge of and attitudes toward the HPV vaccine was constructed using the R statistical programme language package “pheatmap” (https://www.rdocumentation.org/packages/pheatmap/versions/1.0.12/topics/pheatmap accessed on 22 February 2021) based on the percentage of respondents who answered “yes” to a series of questions.

### 2.2. Multivariate Logistic Regression Analysis

A multivariate logistic regression analysis was performed to determine which factors affect knowledge of and attitudes toward HPV and HPV vaccine amongst physicians in Armenia. Using the or_plot function in the R package “finalfit”, nine logistic regression models and forest trees were constructed to determine the odds ratio (OR) and 95% confidence intervals (CI) for physicians answering at random: (1) HPV is relatively uncommon (false); (2) Almost all cervical cancers are caused by HPV (true); (3) HPV is most common in women in their 30 s (true); (4) Cervical cancer is one of the most prevalent cancers among women (true); (5) Most people with genital HPV are symptomatic (false); (6) Genital warts are caused by the same HPV types that cause cervical cancer (false); (7) Sexually active adolescents should be tested before HPV vaccination (false) (8) The HPV vaccine is available for both males and females (true); (9) Men and women who have been diagnosed with HPV should not be given HPV vaccine (false). Factors that were considered in the model were physician speciality, age, and experience.

## 3. Results

A total of 348 participants (90.3% of those eligible) from the 20 randomly selected health facilities (from a total of 36 health facilities located in Yerevan) completed the survey and were included in the final analysis (Figure 1). 

The respondents were predominantly female (95%), reflecting the demographics of the professions (paediatricians, family-practice physicians, gynaecologists, and oncologists). The gender inequality among the respondents (95% female) is not a limitation of this study. According to recent statistics, in Armenia, female doctors are more likely to specialise in paediatrics, family-practice, and gynaecology; hence, the high levels of female respondents in this study. A report published by the Ministry of Health, Armenia, in 2016 shows that 40–60% of physicians were female and, of these, 91.8% were paediatricians, family-practice physicians, and general practitioners; 95.2% of all paediatricians were female [26]. As previous studies of vaccine hesitancy either recorded no significant differences between men and women [27] or have been inconsistent over which gender is more likely to be vaccine hesitant [28,29], a heavily female survey cohort is unlikely to influence perceptions of vaccine hesitancy overall, though this should be tested in future studies if possible. Of those participating in this study, 62% were paediatricians, 16% family-practice physicians, 13% gynaecologists, and 9% were oncologists. The age distribution was 29–60+years of age, with 52% between 45 and 59 years old. Two-thirds (67%) had more than 20 years of experience as doctors.

### 3.1. Knowledge of HPV Transmission, Symptoms, and Disease

In response to some questions, study participants displayed strong awareness of the issues surrounding HPV infection and its related health outcomes; for instance, most knew that cervical cancer is one of the most prevalent types of cancer among women (87.2%) and nearly three-quarters (73.4%) knew that most cervical cancers are caused by the HPV virus (Table 1). Awareness of presentation of the disease was also high, with 81% aware that most people with genital HPV infections are unlikely to display symptoms.

However, 44.4% of the participants were unaware that HPV is a relatively common sexually transmitted infection and 29% of respondents were unaware that the vaccine is available to both females and males. More than half (53.5%) answered that the same types of HPV that cause genital warts also cause cervical cancer, which is incorrect. Only 27% of the respondents were aware that both male and female patients diagnosed with HPV should still be encouraged to receive the vaccine and only a similar proportion (26%) knew that sexually active adolescents do not need to be tested before receiving their HPV vaccinations. Fewer than two thirds (61%) of physicians knew that the HPV vaccine consists of recombinant HPV protein. 

### 3.2. Knowledge Compared by Medical Specialism 

The proportion of correct answers varied among the physicians according to speciality, although the differences were not statistically significant overall (*p* > 0.05). A higher number of correct answers came from paediatricians (65%) than from other groups.

Gynaecologists were more likely than other specialities to answer correctly that HPV is most common in women in their 30s (OR 0.12, *p* = 0.000824), although, as only 40.9% of gynaecologists answered correctly, knowledge was low over all specialties. However, gynaecologists were more likely to answer incorrectly that genital warts are caused by the same strains of HPV, with 65.9% giving an incorrect answer compared with 24.6% of paediatricians (OR 2.17 *p* = 0.0246) (Figure S1). This suggests that the physicians’ level of knowledge is inconsistent across specialties.

For the other questions, differences between specialities were not statistically significant, though paediatricians (74.8%) and oncologists (74.2%) returned a higher rate of correct responses than family practitioners (70.9%) and gynaecologists (68.2%) for the question, “All common cervical cancers are caused by HPV” (*p* = 0.03). There was no significant difference between the specialties in their understanding of whether people with HPV are likely to be symptomatic (they were equally incorrect) (*p* = 0.187), that cervical cancer is one of the most common cancers amongst women, which most answered correctly (*p* = 0.08), and whether or not HPV is a relatively common infection (*p* = 0.643), which just over half of family physicians, oncologists and paediatricians believed to be true and just under half of the gynaecologists (46.5%). 

### 3.3. Knowledge Compared by Age and Length of Professional Service

The proportion of correct answers was significantly different across the age groups (*p =* 0.004). Respondents in the age group 45–59 years old returned a higher rate of correct answers overall. Physicians in the age group under 44 years old were more likely (69.1%) to give a correct answer for the question regarding how common HPV is (Table 1) compared with physicians in the age groups 45–59 and over 60 (OR, 2.56 and 5.26; *p*-value, 0.023637 and 0.000724, respectively) (Figure S1). The 45–59 age group was also more likely to answer correctly that almost all cervical cancers are caused by HPV infection (77.8%) than the lower age group (68.3%, *p* = 0.018) and the over 60 group.

The proportion of correct answers was significantly different when compared across the number of years of service. Respondents with 15–19 years of service answered questions correctly more often (50%) than those who had been in the profession for both a longer (30%) and a shorter time (44.6%) (*p* = 0.003). This difference may be linked to mid-career physicians being more likely to still attend courses and to engage in more educational activities than those nearing the end of their careers, which provides them with more opportunities for keeping up to date with new developments. 

### 3.4. Attitudes towards HPV Vaccination

**Figure 2:**
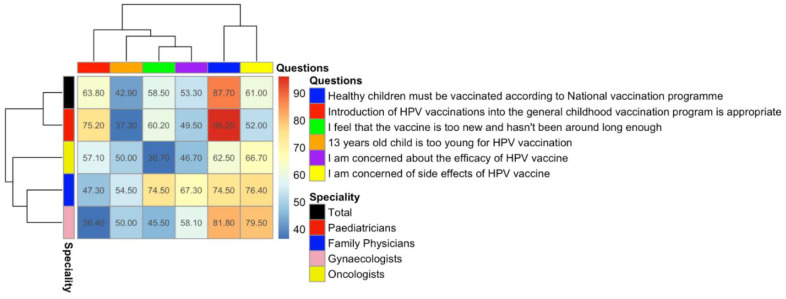
Hierarchy clustering heatmap showing attitudes to HPV vaccine of physicians from different specialities. Number in squares indicates the percentage of who said “yes”.

The survey contained six questions that measured confidence or hesitancy to vaccines in general and the HPV vaccine in particular. The majority of the respondents (87.7%) supported the national vaccination programme for general childhood vaccination but fewer than two-thirds (63.8%) agreed that the introduction of the HPV vaccine into the national vaccination programme was appropriate. More than half (53.3%) expressed concern over the efficacy of the HPV vaccine, 61.0% expressed concerns over its safety, and 58.50% agreed with the statement that the vaccine is “too new and hasn’t been around long enough”. Just under half (42.9%) agreed with the statement that 13 is too young for a child to receive the vaccine (Figure 2).

### 3.5. Support for Vaccination by Specialty

Different specialties displayed statistically significant (*p* < 0.0001) attitudes to the introduction of the HPV vaccine into the national vaccination programme. A higher proportion of paediatricians (75.2%) agreed with it, which was statistically higher than the other groups (oncologists, 57%, family physicians, 47%, and gynaecologists, 36%). The high likelihood of paediatricians to agree with the need for the HPV vaccination programme is consistent with a study from Serbia, in which nearly two-thirds of the paediatricians were willing to recommend the vaccine (60.2%) [30].

The proportion of physicians concerned about the side effects of the vaccine (including the risk of infertility, for which there is no medical evidence) also differed significantly among different specialties (*p* < 0.0001), with a higher percentage of family physicians and gynaecologists showing concern (respectively, 76.4% and 79.5%) than paediatricians and oncologists (52% and 67%). Physicians over 60 years old showed a statistically significantly different level of concern about efficiency and the side effects than those under 60 (respectively, *p* = 0.003, *p* = 0.002) (Table 1 and Table 2).

There was no statistically significant difference in the speciality of physicians who were concerned about the newness of the vaccine (*p* = 0.01) but there was with regard to the age at which it should be given (*p* < 0.0001). Family physicians were more concerned than other specialities that the vaccine was too new (74.5%; nearly double the rate recorded for oncologists at 36.70%) and that a 13-year-old was too young to receive the vaccine (54.5%; against an average across all specialties of 42.90%), suggesting that this speciality is more prone to vaccine hesitancy than others, the reasons for which require further investigation. 

Respondents who were concerned about efficacy were more likely to hold the opinion that the HPV vaccine is too new and “has not been around long enough” (58.8%) (Figure 2). 

The proportion of physicians over the age of 60 who were concerned that 13-year-old females were too young for the vaccination was higher (55.4%) than in younger physicians (39.2%), suggesting that attitudes can differ among demographic groups. The differences in answers to this question among the different age groups were statistically significant (*p* = 0.003). 

In a final question, respondents reported that they had gained information about HPV vaccine from conferences and special events organized by the Ministry of Health, Armenia (72%), specialist literature (63%), colleagues (58%), and self-guided internet searches (57%).

### 3.6. Practice of HPV Vaccination amongst Physicians in Yerevan

No specific questions were asked regarding physicians’ practice in administering the vaccine or not to eligible patients, as information on practice is taken from figures on vaccination coverage in Armenia recorded by existing studies, which show that it is very low compared to other countries [1,2,6,7,8]. Our study sought to explore the reasons for this, which we assume are influenced by the knowledge and attitudes we discuss above. Not having recorded practice specifically is a limitation of the study we would seek to address in further investigations; in particular, it would be valuable to investigate which specific attitudes influence the decisions of individual physicians to administer or not administer the vaccine.

## 4. Discussion

Our study shows that there are knowledge gaps in the understanding of Armenian physicians with regards to some basic aspects of HPV infection and the HPV vaccine. These knowledge gaps affect physicians’ understanding of disease transmission, symptoms, and presentation, as well as who benefits from the vaccine and vaccine safety and efficacy. This is one of the first KAP (knowledge, attitudes and practice) studies to be conducted with regard to physicians’ attitudes towards vaccination in Armenia and its results show that this is a complex area that requires much more nuanced understanding before interventions can be developed. However, it does, suggest that poor knowledge is influencing sub-optimal attitudes and practice.

The level of understanding amongst Armenian physicians (their knowledge) with regard to the mechanisms of disease and vaccination efficacy [31] and benefits is poor. The majority of the physicians knew that cervical cancer is one of the most prevalent types of cancers among women and that it is caused by the HPV virus, but only half of the participants knew that genital warts and cervical cancer are caused by different strains of HPV and one third were unaware that the HPV vaccine is available for both females and males. This suggests that better information about vaccine-controllable diseases and their aetiology may need to be provided as part of vaccination roll-out programmes to ensure that physicians are well informed and their knowledge is up to date.

The particularly low number of correct answers given by the longer-serving group (Table 1) suggests that continued professional training after qualification, to keep one’s skills up to date, may be lacking, although the least experienced group also scored lower than the group with mid-level experience. Further research is required to understand the reasons for this and there may be value in more systematically embedding awareness training as part of continuing professional development programmes for healthcare workers who are likely to be in roles that can influence vaccine take-up or hesitancy.

Almost all respondents were aware of the HPV vaccine but their lack of knowledge impacts on their attitudes to its efficacy and safety and thus its value to patients. Paediatricians were significantly more knowledgeable than other speciality groups, possibly because they were the group most likely to be dealing with the age group that is recommended for vaccination. However, the results show that knowing a vaccine is available does not always correspond to being willing to recommend it, particularly when one is not well informed about it or the disease it can prevent. A total of 62% of physicians were concerned about side effects of the HPV vaccine (although they were not able to specify what any such side effects might be), suggesting that honest discussions around any likely side effects, including their frequency, treatment, and patient outcomes may help to build healthcare practitioner confidence, change their attitudes towards the vaccines, and enable them to pass this confidence on to patients, thus informing their practice around vaccine administration. In addition, 54% of the physicians were worried about the effectiveness of the HPV vaccine, which, again, could be addressed by in-job training and awareness that would help them to change their attitudes towards the value of vaccination and impart greater confidence to patients, who are likely to look to healthcare professionals to advise them.

It is concerning that knowledge gaps exist, despite the fact that almost all respondents reported participating in special events, such as training and seminars, and mentioned specialized literature as one of their main sources of information. This suggests that the current training and methods of information dissemination are ineffective. Further studies are needed to understand the reasons behind these statistics as physicians can act as powerful gatekeepers to vaccines; their level of knowledge influences their attitudes, which, in turn, influence their practice, thus having ramifications for vaccine hesitancy and acceptance (or not) within the communities of patients they attend.

### 4.1. Cultural Assumptions and Misconceptions

We note that cultural assumptions, in particular, influence attitudes and threaten to undermine the scientific basis of vaccine schedules. In Armenia, the routine early childhood immunisation coverage is estimated at 93–97% of all children and physicians strongly support vaccination programmes amongst this age group [17]. Our study suggests that, whilst early childhood vaccinations are strongly supported by most of the physicians surveyed (87.70%), attitudes are different towards the HPV vaccine; fewer are comfortable with the inclusion of the HPV vaccine into the national vaccination programme (63.80%). The physicians had a tendency to associate HPV vaccination with the onset of sexual activity and felt that support for the vaccination of young girls was a tacit acceptance of early sexual activity. This attitude was more frequently found among physicians over 60 years old, suggesting that vaccine hesitancy is more complex than a binary “yes/no”; a “no” for one age cohort may become a “yes” for another. Controversies among health professionals relating to the inclusion of HPV in national immunization schedules has also been described in Spain, where 89% of health professionals were aware of the relationship between HPV infection and cervical cancer but 65.7% resist its introduction into vaccine schedules, arguing that there are no data on its long-term effectiveness [28]. 

However, the age of vaccine administration is not, in fact, determined by the age at which sexual activity is likely to begin. According to the WHO, the recommended priority age for HPV vaccination is 9–13 years old because this is the age at which the immune system response is highest and produces the necessary level of antibodies and immune-system memory to protect against HPV infection for life, not because this is the age at which children may become sexually active. It is important for preteens to get all three doses long before any sexual activity begins [32]. This shows a direct link between level of knowledge and cultural attitudes and beliefs, which intersect with scientific evidence to build acceptance or resistance to vaccination. Vaccine confidence or hesitancy can be based on sociocultural factors as well as medical science; thus, it is important to ensure that attitudes based on incomplete knowledge, that can impede vaccination, are addressed through improving knowledge. 

The Armenian physicians expressed considerable concern over the newness of the Gardasil vaccine (from 36.7% amongst oncologists to 74.5% in family physicians; 58.5% across all specialities). This attitude may require targeted education to help healthcare providers understand the development and approvals process for these new vaccines in order to give assurance that the procedures have been conducted safely [24], thus giving medical staff the confidence to reassure patients that new vaccines are safe. By paying more attention to how a divide between the scientific basis for the decision to vaccinate and the cultural assumptions against vaccination at this particular age influences physicians’ attitudes, we can start to explore how such attitudes can be changed. It also illustrates that, by not being aware of such assumptions and failing to take time to address them in awareness information and training given to professionals responsible for administering the vaccine, vaccine confidence can be undermined by misconceptions and cultural perceptions that may prove harder to dispel later on, preventing the practice of appropriate vaccination. 

### 4.2. Vaccine Hesitancy May Not Be Universal

The different levels of confidence in the national childhood vaccination programme compared with the introduction of the HPV vaccination highlight that individual vaccines need to be considered independently of one another, as attitudes may differ between one vaccine and another, or one context and another. Vaccine hesitancy may not be universal to all vaccines nor have the same underlying concerns with regard to each vaccine. Understanding these attitudes more fully would benefit from further research to help determine how different forms of hesitancy can be effectively challenged, how they influence and impact on practice, and what steps might be taken in advance to stop such hesitancy from becoming widespread within certain communities. Refusal of one vaccine does not necessarily point to complete vaccine denial. 

Whilst we appreciate that HPV and Gardasil are one specific case, we do believe that the above findings highlight issues that are likely to be generalizable to other vaccines and to be transferable to attitudes towards vaccines more widely than the Gardasil vaccine and HPV alone. Firstly, they identify a spectrum of vaccine hesitancy [33], in which vaccine acceptance or refusal is not absolute but dependent on complex factors that may be unique to the time, context, and even the individual physician—for example, the newness of the vaccine, the age of the patient to whom it is due to be administered, and misconceptions about the vaccine’s purpose amongst healthcare workers. Awareness of this complexity is also likely to be needed to inform vaccination strategies for COVID-19, another vaccine for which high levels of hesitancy have been observed in Armenia, some also linked to the newness of the vaccine in particular. As of 24 May 2021, only 26,562 COVID-19 vaccines had been administered in the country, covering just 0.8% of the population, although vaccine doses were readily available [34]. Despite the widespread and growing prevalence of COVID-19 in Armenia, a nationwide poll by the International Republican Institute’s (IRI) Centre for Insights in Survey Research, published on 28 May 2021, recorded that 71% of Armenians (75% in Yerevan) would not get vaccinated against COVID-19 if vaccines were made available [7], whilst a different survey, conducted by Gallup International in April 2021 [8], also put vaccine hesitancy at above 51.9%, with the exact rate differing considerably between the UK-developed AstraZeneca (9.2%) and the Russian-developed Sputnik V (75.8%) vaccines. The vaccines for COVID-19 are, like those for HPV, new; the Armenian physicians who expressed concern over the newness of the Gardasil vaccine (from 36.7% amongst oncologists to 74.5% in family physicians; 58.5% across all specialities) may well also be concerned about vaccines for COVID-19, which are not only new but have been developed more quickly than usual. Secondly, though linked to the first point, as with the HPV vaccine, Armenians who are generally accepting of most vaccines can have concerns over others, meaning that a population that is highly vaccinated against existing diseases may not automatically be receptive to public health messaging campaigns for others; only 13% of Armenians who reported they would not take a COVID-19 vaccine claimed they do not get vaccines in general [7]. Both these points suggest that there is considerable room for confidence-building around newly introduced vaccines, but that this will be more difficult if medical staff, to whom patients look for information, are also hesitant. However, we must be careful, in assuming that findings from a KAP study on one vaccine can be applicable to another; thus, how the findings from this study relate to confidence (or not) in other vaccines must be examined further. We present this as a basis from which further investigation should begin.

Further studies could also usefully determine the extent to which physicians’ poor understanding, misconceptions, and hesitancy may influence their decision to administer vaccines and their ability to sufficiently inform patients of vaccine benefits. 

### 4.3. Limitations

Our study is represented by health facilities (and physicians) from Yerevan only, for a single vaccine, and during a specific period of time. Therefore, the findings may not be generalizable to the whole of Armenia, or to physicians in other countries, or to different vaccination programmes. Knowledge, attitudes, and practice of respondents working in medical facilities located out of Yerevan and with different vaccines may vary from our study results. The assumptions we made about the generalisability of these findings to vaccine confidence in general are assumptions only and need to be tested in further research studies. 

## 5. Conclusions

This study shows that the concerns and worries that influence negative attitudes towards vaccines and which may prevent physicians from recommending vaccination are complex. Reasons may differ for different diseases and may be influenced by different underlying factors, all of which suggest the need for stronger vaccine-confidence awareness training that helps physicians—and, by extension, the patients they advise—to navigate these concerns by providing up-to-date and comprehensive information that will enhance confidence and dispel unwarranted concerns. The surveyed healthcare workers were generally not hesitant to the concept of vaccines, more to uncertainties about aspects of vaccine development, efficacy, or side effects that can be answered with easier access to accurate and up-to-date information. The role of the media and its portrayal of vaccination development may also have some influence in this process and should be considered within future research programmes. 

Differences in the approach of different physicians in Armenia to HPV vaccination was evident in this study, with different approaches noted based on their age, length of service, speciality, and cultural assumptions. Overall, however, the study merely highlights gaps in our understanding of vaccine hesitancy and how this may vary between different vaccines and for the prevention of different diseases; it does not address them or offer interventions. In this regard, priority consideration should be given to further studying and, in the short term, to developing a programme of continuous education for Armenian healthcare professionals on all aspects of vaccination development, safety, efficacy, and societal value, both for HPV infections and for other newly developed vaccines, ensuring such knowledge is not taken for granted when introducing new vaccines.

## Figures and Tables

**Table 1:** Knowledge of HPV of physicians practicing different specialist areas.

Physicians’ Characteristic	HPV Is a Relatively Uncommon Sexually Transmitted Infection			Almost All Cervical Cancers Are Caused by HPV Infection			Most People with Genital HPV Infections Are Symptomatic			Genital Warts Are Caused by the Same HPV Types That Cause Cervical Cancer		
	T	F	DK	T	F	DK	T	F	DK	T	F	DK
**Specialty**												
Family physicians	38.9	53.7	7.4	70.9	20	9.1	20.4	75.9	3.7	43.6	43.6	12.8
Paediatricians	36.9	58.3	4.9	74.8	20	5.2	11.8	80.4	7.8	65.2	24.6	10.1
Gynaecology	48.8	46.5	4.7	68.2	31.8	0:0	9.1	90.9	0.0	22.7	65.9	11.4
Oncology	35.5	51.6	12.9	74.2	12.9	12.9	16.7	80	3.3	32.3	58.1	9.7
**Age**												
Less than 44	28.4	69.1	2.5	68.3	28.0	3.7	10.1	87.3	2.5	45.0	43.8	11.2
45–59	39.0	55.9	5.1	77.8	18.9	3.3	11.9	82.4	5.7	56.5	33.9	9.6
Older than 60	48.1	40.7	11.1	68.7	18.1	13.2	18.3	73.2	8.5	54.8	32.1	13.1
**Experience (Years)**												
Less than 14	33.8	63.1	3.1	67.7	30.8	1.5	6.2	90.6	3.1	38.5	44.6	16.9
15–19	42.9	57.1	0.0	83.7	14.3	2.0	12.2	87.8	0.0	47.9	50.0	2.1
More than 20	38.9	52.9	8.1	73.5	18.6	7.9	16.0	76.3	7.7	59.2	30.0	10.8

T, true; F, false; DK, do not know.

**Table 2:** Knowledge of HPV. Comparison between physicians practicing different specialist areas.

True/False Statement	% Correct Total	% Correct Family Physicians	% Correct Paediatricians	% Correct Gynaecologists	% Correct Oncologists	Chi-Square	*p*-Value
HPV is relatively uncommon.	55.6	53.7	58.3	46.5	51.6	5.653	0.643
Almost all cervical cancers are caused by HPV.	73.4	70.9	74.8	68.2	74.2	18.436	0.03
HPV is most common in women in their 30s.	15.1	10.9	9.5	40.9	22.6	36.058	0.000
Cervical cancer is one of the most prevalent cancers among women.	87.2	80	89	88.6	86.7	11.288	0.08
Most people with genital HPV infections are symptomatic.	81.1	75.9	80.4	90.9	80	8.766	0.187
Genital warts are caused by the same HPV types that cause cervical cancer.	35.7	43.9	24.6	65.9	58.1	40.452	0.000
The HPV vaccine is available for both males and females.	71	73.6	70.2	72.1	65.5	6.575	0.362
Sexually active adolescents should be tested for HPV before vaccination.	26.2	22.2	31.1	13.6	24.1	1.952	0.924
Men and women who have been diagnosed with HPV should not be given HPV vaccine.	27.2	24.5	28	25	31	1.952	0.952

## Data Availability

The data used for analyses is presented in the paper.

## Supplementary Materials

The following is available online at https://www.mdpi.com/article/10.3390/vaccines9101188/s1, Figure S1, Multivariate logistic regression analysis and forest plot of respondents’ awareness of HPV and it’s vaccine in Armenia, OR = odd’s ratio, CI = confident interval.
