# Supplementary material for: Knowledge, Attitude, and Practice of Physicians Regarding Vaccinations in Yerevan, Armenia: A Case Study of HPV
Source: Vaccines (Basel). 2021 Oct 15;9(10):1188. doi: 10.3390/vaccines9101188 (PMC8540740; doi:10.3390/vaccines9101188)
Supplement: Supplementary file 1 [file vaccines-09-01188-s001.zip › vaccines-1363606-supplementary.pdf]

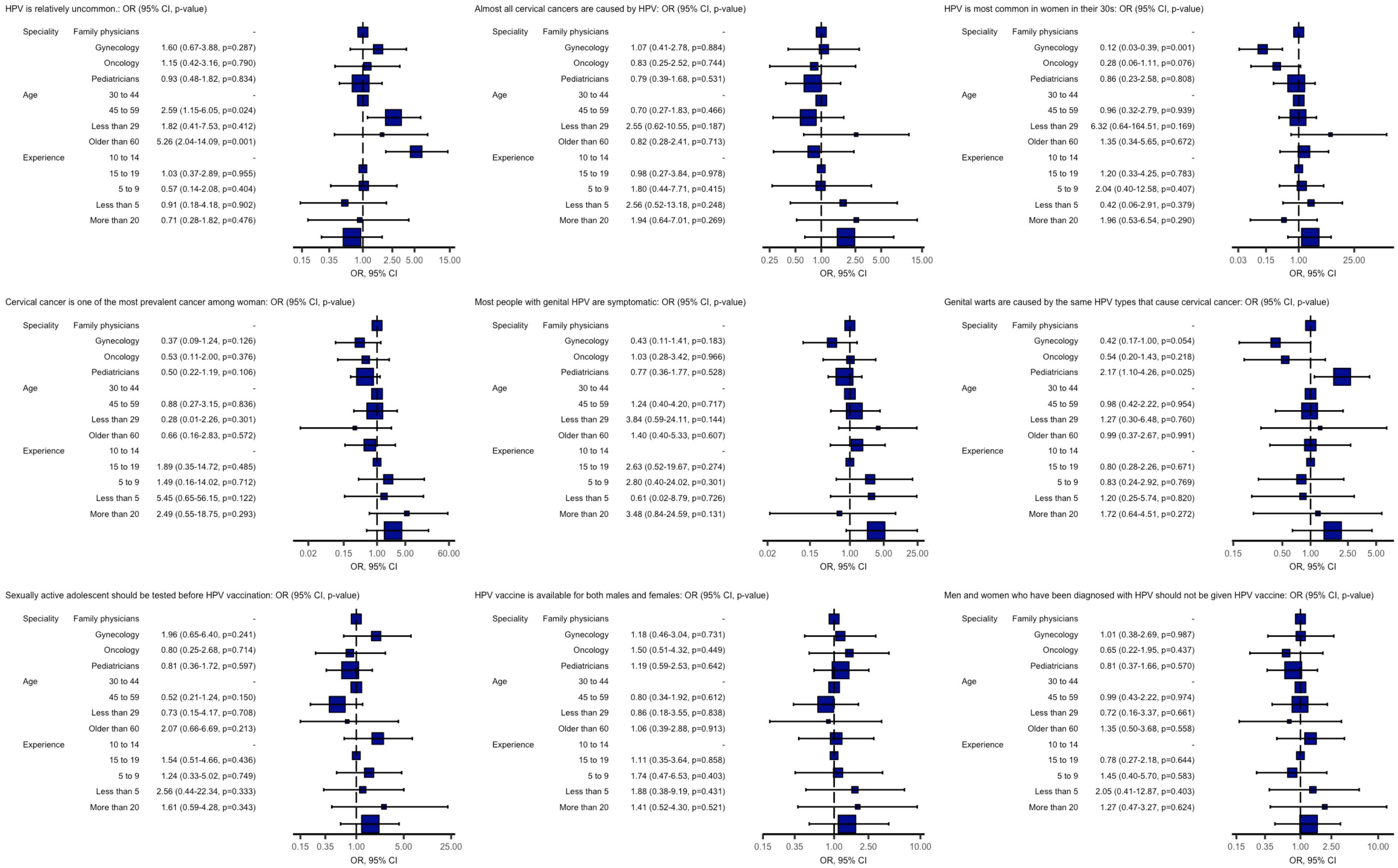

Figure S1: Multivariate logistic regression analysis and forest plot of respondents’ awareness of HPV and it’s vaccine in Armenia, OR = odd’s ratio, CI= confident interval.
